# Therapeutic potential of Chinese herbal medicine for coronary heart disease patients with cerebral ischemic stroke: a systematic review and meta-analysis

**DOI:** 10.3389/fphar.2025.1578783

**Published:** 2025-07-16

**Authors:** Rui Du, Yuhan Ao, Yang Wang, Zhihui Chen, Guanghui Liu, Mingxue Zhang

**Affiliations:** ^1^ First Clinic College, Liaoning University of Traditional Chinese Medicine, Shenyang, China; ^2^ Department of Senile Disease, Affiliated Hospital of Liaoning University of Traditional Chinese Medicine, Shenyang, Liaoning, China; ^3^ Basic Medical College, Liaoning University of Traditional Chinese Medicine, Shenyang, China; ^4^ Basic Department of Science and Technology Management, Affiliated Hospital of Liaoning University of Traditional Chinese Medicine, Shenyang, Liaoning, China; ^5^ Department of Encephalopathy Rehabilitation, Affiliated Hospital of Liaoning University of Traditional Chinese Medicine, Shenyang, Liaoning, China; ^6^ Department of Cardiovascular Medicine, Affiliated Hospital of Liaoning University of Traditional Chinese Medicine, Shenyang, Liaoning, China

**Keywords:** cerebral ischemic stroke, Chinese herbal medicine, systematic review and meta-analysis, coronary heart disease, cardiovascular and cerebrovascular chronic diseases

## Abstract

**Objective:**

This research sought to demonstrate potential therapeutic strategies for coronary heart disease (CHD) patients with cerebral ischemic stroke (CIS) by rigorously evaluating the efficacy and safety of Chinese Herbal Medicine (CHM) through meta-analysis.

**Methods:**

A broad search approach was applied to obtain pertinent articles from both domestic and international databases, covering publications up to 31 December 2024. Using RevMan software (Version 5.4), a systematic review and meta-analysis were conducted to assess the efficacy and safety of CHM in treating CHD patients with CIS.

**Results:**

In the meta-analysis, 18 trails were analyzed, encompassing 2,202 patients in total. The aggregated findings indicated that the utilization of CHM improved the overall effective rate, ECG performance and TCM scores significantly. Furthermore, the CHM therapy demonstrated significant improvements in LVEF, MMSE, and NIHSS. Additionally, the CHMs therapy positively influenced lipid profiles, specifically TC, TG, LDL-C, and HDL-C. Notably, the application of CHM during the intervention was particularly effective in reducing blood viscosity, fibrinogen and platelet aggregation. Importantly, the CHM therapy was found to provide comparable safety profile to that of conventional western medicine treatment (WM) alone.

**Conclusion:**

The CHM demonstrated superior efficacy in the management of CHD patients with CIS. Concurrently, the CHM showed potential for improving neurological damage, lipid profiles, and positively affecting hemorheological parameters, all while minimizing the risk of adverse effects. Even so, because of the limitations in study quality and the potential for reporting bias, it is crucial that these findings require to be further validated through rigorous, large-scale, and high-quality RCT in future research.

## 1 Introduction

The cardiovascular and cerebrovascular disorders remains a major challenge to the health system worldwide, especially the co-morbidity of cardiovascular and cerebrovascular conditions is becoming increasingly prevalent (Sposato et al., 2020; Gu et al., 2019). The latest data from The Global Burden of Disease Study pointed out that cardio-cerebrovascular disease are currently affecting nearly 523 million people worldwide and accounted for 18.6 million deaths (GBD, 2019 Stroke Collaborators, 2021). Cardio-cerebrovascular disease is the top cause of mortality among residents in China with studies indicating a steady increase in its incidence and prevalence, among which CHD and CIS account for the highest proportion (Chen et al., 2018). The National Center for Cardiovascular Diseases has reported that there are approximately 13 million and 11.39 million patients with CIS and CHD, respectively (National Health Commission, 2021). Both conditions share a common pathological basis and similar pathogenesis, often leading to their concurrent occurrence and interaction. A retrospective survey involving 1,802 elderly patients revealed that 32% of those with CHD also suffered from CIS, while 56% of CIS patients had concurrent CHD (Yang, 2020). Furthermore, research utilizing the JRoad-DPC and Trine TX database indicates that patients with both cerebrovascular and cardiovascular diseases have significantly higher five-year mortality and poor prognosis rates than those with just one of these conditions (Nakai et al., 2022). These findings underscore the substantial prevalence of CHD complicated by CIS, as well as the high rates of recurrence, disability, and mortality associated with this comorbidity, highlighting the significant challenges faced in its prevention and treatment.

Contemporary advancements in modern medicine have markedly improved the treatment for CHD patients with CIS, significantly reducing both recurrence rates and mortality (Tsao et al., 2023). The predominant strategy for managing CHD with concurrent CIS involves a multifaceted approach targeting various risk factors, including blood pressure regulation, cardiovascular enhancement, and neural nourishment (Wang et al., 2022). Nevertheless, the complexity of multi-drug treatments can increase the likelihood of patients not following their prescriptions, and extended use of medications might cause negative effects like stomach issues and liver problems (Zhu et al., 2022). Given the problems associated with current therapeutic strategies, healthcare professionals are increasingly investigating the potential of CHM as a complementary treatment modality.

The patient-centered, holistic, and multi-dimensional approaches employed in traditional Chinese medicine (TCM) have shown distinct advantages in managing complex conditions such as CHD complicated by CIS (Marín-Peñalver et al., 2016). The condition known as ‘Xiongbi complicated with Zhongfeng’ in TCM, identified by its clinical signs and features, is initially recorded in the ancient Chinese medical work, the Yellow Emperor’s Classic of Internal Medicine. In recent years, increasing preclinical and clinical evidence has supported the efficacy of CHM in managing CHD patients with CIS. Nonetheless, the majority of published clinical trials are limited in scope, predominantly comprising small-scale, single-center studies that lack comprehensive systematic evaluation and review of clinical interventions. Therefore, rigorous evidence-based research is essential to validate the efficacy and safety of CHM in the management of CHD complicated by CIS.

This study, building on the earlier mentioned background, seeks to perform an extensive review of national and international literature to objectively assess the clinical benefits and safety of CHM for patients suffering from CHD complicated by CIS, with the goal of elucidating potential clinical strategies.

## 2 Materials and methods

This protocol has been registered with the INPLASY platform under the registration number INPLASY202510112.

### 2.1 Outline of literature retrieval

A detailed investigation of numerous databases was undertaken to assess CHM therapeutic remedies for CHD patients with CIS. The databases referenced were PubMed, EMBASE, Web of Science, and the Cochrane Library, in addition to CNKI, Wanfang, VIP, and CBM. The data retrieval encompassed the complete temporal range of all databases up to 31 December 2024, without restrictions on language, participant condition, or publication year. In the current search strategy, keywords and MeSH terms were systematically combined, with a specific emphasis on “CHD complicated by CIS” and “CHM” Additionally, the search included interventions and diseases relevant to the study subjects, such as TCM, herbal medicine, Chinese medicine, and CHD complicated with stroke, among others. To ensure thoroughness and mitigate any potential omissions, a manual review of journal literature was also performed. Supplementary Material S1 provides detailed descriptions of the search strategy for each database.

### 2.2 Inclusion criteria

(1) Participant Characteristics. The study included participants without age, gender, or race limitations. Individuals diagnosed with CHD and CIS according to international diagnostic criteria or a clearly defined standard were involved.
(2) Intervention Types. The experimental group in this study received CHM, in addition to receiving the same conventional Western medicine treatment as the control group. No restrictions were placed on the amount or length of the treatment provided.
(3) Types of Comparison: Conventional western medicine treatments (WM) with proven effectiveness in improving CHD and CIS. In the studies reviewed, there were no variations in the specifications or dosages of WM between the control and experimental group.
(4) Types of Outcomes. The primary endpoints were the overall effective rate (Overall effective rate = Number of cured and improved patients/total number of patients) and adverse reactions, with secondary outcomes including improvement of electrocardiography (ECG), TCM score, LVEF, MMSE score, NIHSS score, TC, TG, LDL-C, HDL-C, blood viscosity, FIB,and platelet aggregation. A minimum of one result has been reported from every article included in the review.
(5) Study Design Categories: This investigation covered all trails documenting the utilization of CHM for treating CHD patients with CIS, regardless of language or publication status.

### 2.3 Exclusion criteria

The following were outlined as the exclusion criteria: (1) Studies that were not clinical trials or involved animal subjects were excluded. (2) Studies in which the control group employed CHM modalities, such as Chinese patent medicine, acupuncture, herbal extracts, and analogous interventions, were not considered. (3) Studies identified as duplicate publications or containing redundant clinical data were excluded. (4) Studies for which original data were inaccessible or could not be extracted, despite efforts to contact the authors, were also excluded. (5) Studies where the outcome effect was ambiguous due to incomplete data, unclear reporting of outcomes, or inappropriate statistical methods were included.

### 2.4 Baseline characteristics of studies

A team of two independent researchers extracted data from these studies. To improve efficiency, various variables, including first authors, publication dates, countries, study designs, sample sizes, mean ages, genders, intervention measures, and follow-up durations, were systematically organized in a study-specific Excel spreadsheet. The data were then subjected to cross-validation before being imported into Review Manager. Following the Cochrane Handbook for Systematic Reviews guidelines, the potential for bias in each study included in the analysis was evaluated with the Risk of Bias (RoB) tool version 1.0. If there were disagreements, a third reviewer was brought in to help reach an agreement.

### 2.5 Statistical analysis

Review Manager (version 5.4) together with Stata software (version 17.0) was employed for all analyses. The odds ratio (OR) was used for binary variables, while the mean difference (MD) or standardized mean difference (SMD) was employed for continuous variables, depending on the measurement units. The findings were presented with 95% confidence intervals. Chi-square statistics were used to evaluate heterogeneity, which was considered present if the P-value was below 0.1 and the I^2^ statistic exceeded 50%, leading to the application of a random-effects model. Alternatively, the use of a fixed-effects model was warranted when the P-value was at least 0.1 and the I^2^ statistic did not exceed 50% (Higgins and Thompson, 2002). By systematically excluding individual studies, a sensitivity analysis performed to determine how low-quality studies affect the stability and robustness of the meta-analysis. To determine publication bias, Begg’s funnel plot and Egger’s test were employed. Descriptive statistics was performed using Excel.

### 2.6 Certainty assessment of evidence

For the weighted averages of different effect estimate, two reviewers independently assessed the certainty of evidence using the Grading of Recommendations, Assessment, Development, and Evaluations (GRADE) system. The certainty of evidence was categorized as high, moderate, low or very low. At the beginning of the assessment, RCTs started as high certainty and could be downgraded due to five reasons: risk of bias, imprecision, inconsistency, indirectness, and publication bias; OBs started as low certainty, which could be downgraded due to five reasons same as RCTs, and upgraded due to three reasons: large magnitude of an effect, dose-response gradient, and effect of plausible residual confounding.

## 3 Results of meta-analysis

### 3.1 Overview of literature retrieval

**FIGURE 1:**
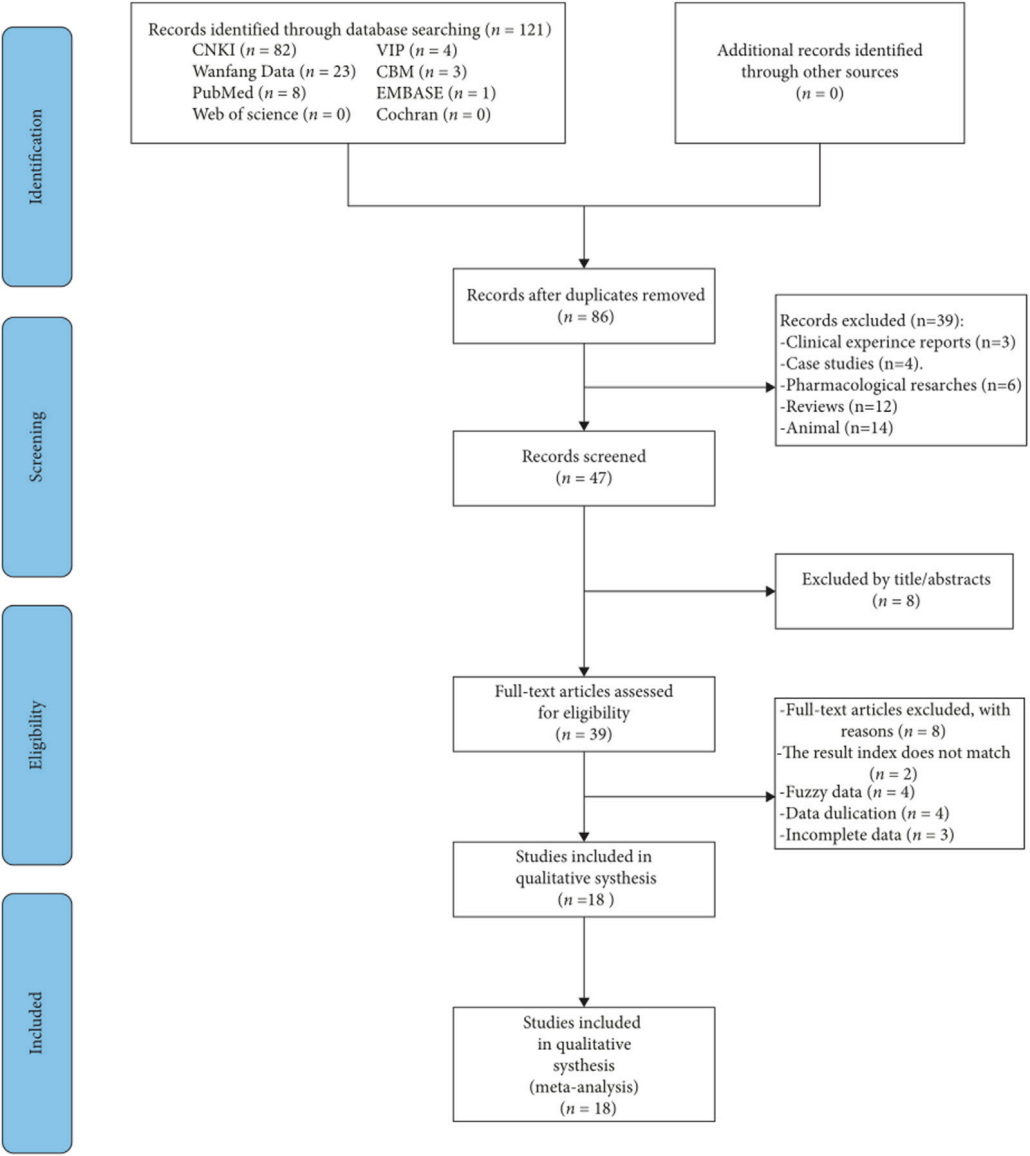
Outline of the study selection procedure.

The review began with 121 articles, of which 18 met the criteria for inclusion (Yang, 2015; Wu, 2022; Liao et al., 2016; Zhang et al., 2008; Wang et al., 2018; Chang et al., 2024; Sun, 2018; Li H et al., 2016; Zeng et al., 2022; Yu, 2024; Wen, 2013; Yuan, 2023; Wu and Li, 2023; Feng, 2023; Ma et al., 2022; Liang, 2024; Liu and Che, 2020; Song et al., 2021). Figure 1 provided details of the search process.

### 3.2 Description of study characteristics and bias risk

**FIGURE 2:**
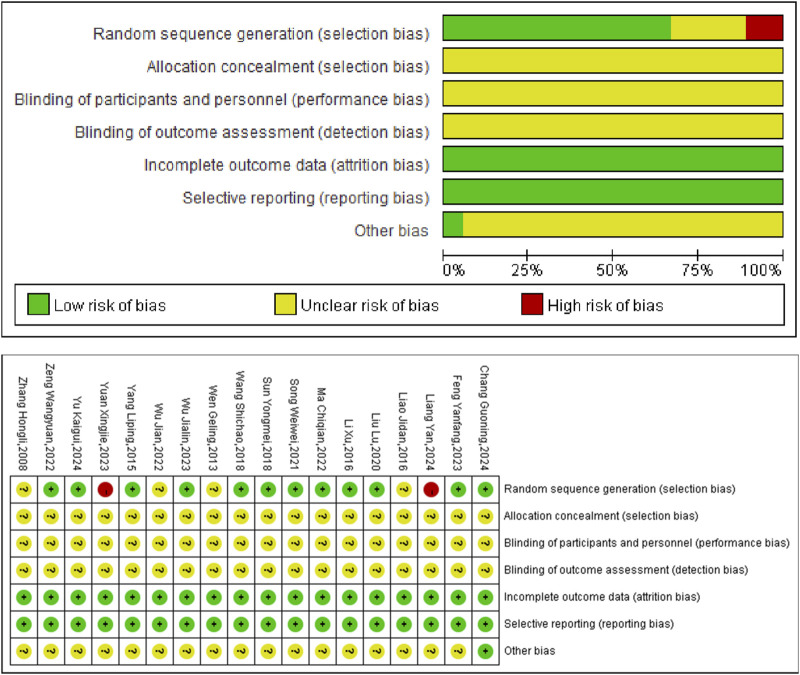
Included studies’ risk of bias plot.

The present meta-analysis incorporated 18 studies comprising a total of 2,202 individuals, with 1,092 cases in the experimental group and 1,110 cases in the control group (Table 1). The components of Chinese herbal medicine used in included studies were displayed in Supplementary Material S2. The risk of bias was assessed using Rev Man 5.4 software (Figure 2).

**TABLE 1:** Summary of included studies.

Author (year)	Gender (M/F)		Course of CHD (y)	Course of CI (d)	Mean age (y) E/C	Intervention		Duration (months)	Outcomes
	E	C	E/C	E/C		E	C		
Yang (2015)	34/25	33/25	6.37 ± 1.01/6.14 ± 1.21	NR	52.38 ± 5.08/52.11 ± 5.01	Maixuekang + C	Conventional treatment (Calcium antagonists, beta blockers, angiotensin converting enzyme inhibitors, etc.) and clopidogrel hydrogen sulfate	3	②⑥⑦⑧⑨
Wu (2022)	26/19	25/20	4.64 ± 1.17/4.25 ± 1.12	NR	64.48 ± 4.59/64.23 ± 4.64	Danhong injection + C	Conventional treatment (Beta blockers, aspirin, isosorbide dinitrate tablets, atorvastatin) and flunarizine capsules	0.5	①⑦⑨
Liao et al. (2016)	120/180	173/127	NR	NR	76.8/77.5	Danhong injection + C	Conventional treatment (Baiaspirin tablets, metoprolol sustained-release tablets, atorvastatin calcium tablets) and injection of low molecular weight heparin calcium injection	NA	②⑦⑧
Zhang et al. (2008)	20/10	18/12	NR	47/45	57/54	Danhong injection + C	Sodium ozagrel for injection, phosphatidylcholine, aspirin	NA	①⑥⑦⑨
Wang et al. (2018)	30/24	29/25	NR	26.63 ± 14.84/25.75 ± 15.42	64.46 ± 8.97/63.38 ± 9.02	Deng Tietao Foot Bath Prescription + C	Conventional treatment (antiplatelet aggregation, blood pressure reduction, blood lipid reduction and treatment of CHD)	1	①⑤⑥⑦
Chang et al. (2024)	22/21	25/18	NR	NR	64.87 ± 4.99/65.21 ± 5.02	Maixuekang + C	Conventional treatment (Oxygen inhalation, improvement of microcirculation, nourishment of the brain and nerves, reduction of intracranial pressure, maintenance of electrolyte balance), rosuvastatin calcium tablets, butylphthalein soft capsules	1	①②③④⑤⑥
Sun (2018)	15/15	16/14	NR	NR	60.22 ± 11.89/60.61 ± 12.17	Huatan Xiaoshuan decoction + C	Conventional treatment (Antiplatelet aggregation, lipid regulation, blood pressure control, nitrate ester preparations, nourishing brain cells)	0.5	⑥
Li H et al. (2016)	19/13	18/15	NR	NR	65.50 ± 1.40/66.03 ± 1.32	Huayu Qutan decoction + C	Conventional treatment (Aspirin enteric coated tablets, atorvastatin calcium tablets, isosorbide mononitrate sustained-release tablets)	1	①③⑥⑦⑨
Zeng et al. (2022)	41/23	43/21	2.3 ± 0.9/2.4 ± 0.8	9.3 ± 0.8/9.5 ± 0.7	65.4 ± 5.8/65.8 ± 5.5	Huoxue Huatan recipe + C	Conventional treatment (Aspirin enteric coated tablets, atorvastatin calcium tablets, isosorbide mononitrate sustained-release tablets) and clopidogrel hydrogen sulfate	1	①④⑤⑥
Yu (2024)	20/19	22/17	NR	NR	75.02 ± 2.54/75.23 ± 2.77	Huoxue Huatan recipe + C	Conventional treatment (Aspirin enteric coated tablets, atorvastatin calcium tablets, isosorbide mononitrate sustained-release tablets)	1	④
Wen (2013)	25/21	25/19	NR	NR	80.1/79.2	Self-made Huoxue Tongyu Decoction + C	Conventional treatment	1	②
Yuan (2023)	20/22	26/14	13.45 ± 3.45/12.64 ± 3.36	NR	68.76 ± 4.24/67.28 ± 3.28	Naoxintong tablets + C	Conventional treatment + Sodium ozagrel for injection	1	①⑧⑨
Wu and Li (2023)	19/13	15/17	NR	16.18 ± 2.25/12.64 ± 3.36 (hours)	63.15 ± 5.88/62.74 ± 6.05	Shenmai injection + C	Conventional treatment (Antiplatelet aggregation, thrombolysis, lipid-lowering, anticoagulation, vasodilation, plaque stabilization, intracranial pressure reduction, oxygen therapy) and Edaravone Injection	0.5	①⑤⑥⑧
Feng (2023)	21/19	23/17	2.10 ± 0.52/2.08 ± 0.55	NR	58.91 ± 5.33/58.95 ± 6.04	Ginkgo diterpenoid lactone meglumine injection + C	clopidogrel hydrogen sulfate	3	①③⑧⑨
Ma et al. (2022)	59/48	56/51	2.08 ± 0.38/2.07 ± 0.28	NR	59.47 ± 4.97/59.07 ± 4.28	Ginkgo diterpenoid lactone meglumine injection + C	clopidogrel hydrogen sulfate	3	①③⑤⑧⑨
Liang (2024)	32/18	30/20	NR	15.09 ± 2.92/15.42 ± 2.83 (hours)	65.63 ± 2.42/65.69 ± 2.48	Ginkgo diterpenoid lactone meglumine injection + C	clopidogrel hydrogen sulfate	0.5	①③⑤⑥⑧
Liu and Che (2020)	71/55		NR	NR	56.37 ± 11.03	Compound Danshen Dripping Pills + C	Conventional treatment (control of blood glucose, blood pressure, and blood lipids) + vinpocetine injection	0.5	①④⑥⑧
Song et al. (2021)	70/54		7.98 ± 1.30	18.18 ± 4.16 (hours)	61.18 ± 6.64	Salvianolic acid injection + C	Conventional treatment (antiplatelet aggregation, lipid regulation, coronary artery dilation, oxygen therapy, control of cerebral edema, reduction of intracranial pressure, improvement of microcirculation, maintenance of water and electrolyte balance, and protection of cerebral nerves) + atorvastatin calcium tablets	0.5	①⑥⑦

E, Experimental group; C, Control group; NR, Not reported; Outcome, ①Overall effective rate; ②ECG; ③TCM, scores; ④LVEF; ⑤MMSE, score; ⑥NIHSS, score; ⑦Blood lipid indicators; ⑧Hemorheology indicators; ⑨Adverse reaction.

### 3.3 Results of meta-analysis

#### 3.3.1 Overall effective rate

**FIGURE 3:**
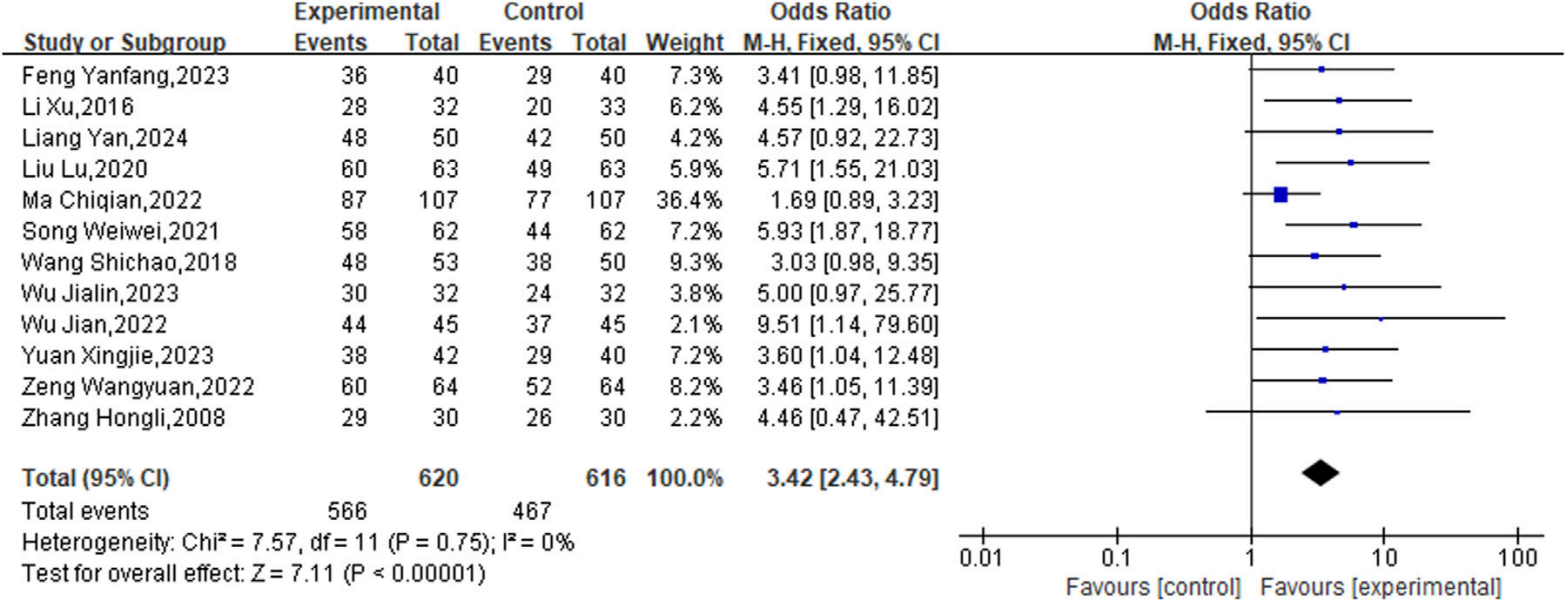
Meta result of Overall effective rate.

The current analysis encompassed 12 studies involving a total of 1,236 patients, employing a fixed-effects model for statistical evaluation (P = 0.75, I^2^ = 0%). The findings showed that the experimental groups notably enhanced the overall effective rate relative to the control groups (OR = 3.42, 95% CI [2.43, 4.79], P < 0.00001) (Figure 3).

#### 3.3.2 ECG performance

**FIGURE 4:**
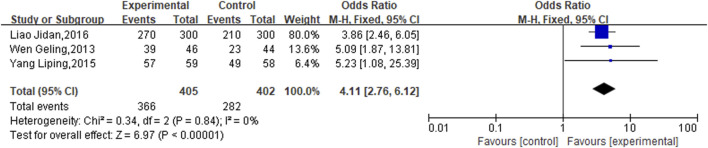
Meta result of ECG performance.

3 studies specifically addressed ECG performance, and due to the observed homogeneity, a fixed-effects model was deemed appropriate (*P* = 0.84, *I* ^*2*^ = 0%). In CHD patients with CIS, the experimental groups significantly enhanced ECG scores relative to the control groups (OR = 4.11, 95% CI [2.76,6.12]; P < 0.00001) (Figure 4).

#### 3.3.3 TCM scores

**FIGURE 5:**
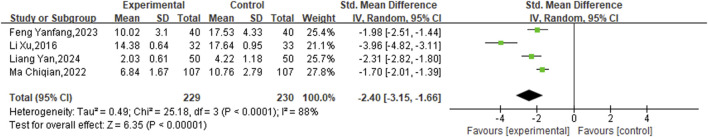
Meta result of TCM scores.

The analysis of four studies, which included 459 patients, was conducted using a random-effects model after a heterogeneity test (P < 0.0001, I^2^ = 88%), as illustrated in Figure 5. Findings from the meta-analysis demonstrated that TCM scores decreased more in the experimental groups than in the control groups (SMD = −2.40, 95%CI [−3.15, −1.66], P < 0.00001).

#### 3.3.4 Left ventricular ejection fraction (LVEF)

**FIGURE 6:**
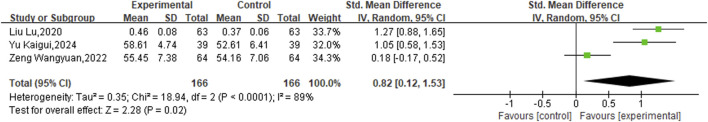
Meta result of LVEF.

The analysis of LVEF included three studies with a combined total of 332 patients, utilizing a fixed-effects model for statistical evaluation (P = 0.80, I^2^ = 0%). The findings demonstrated that the experimental groups led to a statistically significant improvement in LVEF compared to the control groups (SMD = 0.82, 95% CI [0.12, 1.53]; P = 0.02) (Figure 6).

#### 3.3.5 Mini-mental state examination (MMSE) score

**FIGURE 7:**
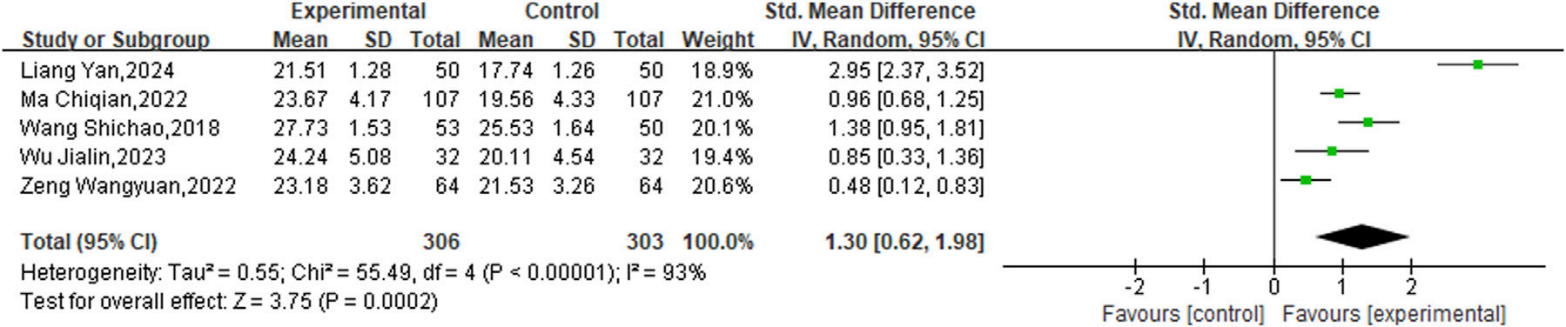
Meta result of MMSE.

Furthermore, five eligible studies encompassing a total of 609 patients, allocated into two distinct study groups, were incorporated to evaluate the MMSE scores. Given the observed heterogeneity (P < 0.00001, I^2^ = 93%), a random-effects model was employed for this analysis. The results clearly indicated that the experimental groups led to a significant enhancement in MMSE scores relative to the control groups (SMD = 1.30, 95% CI [0.62,1.98], P = 0.0002) (Figure 7).

#### 3.3.6 National institute of health stroke scale (NIHSS) score

**FIGURE 8:**
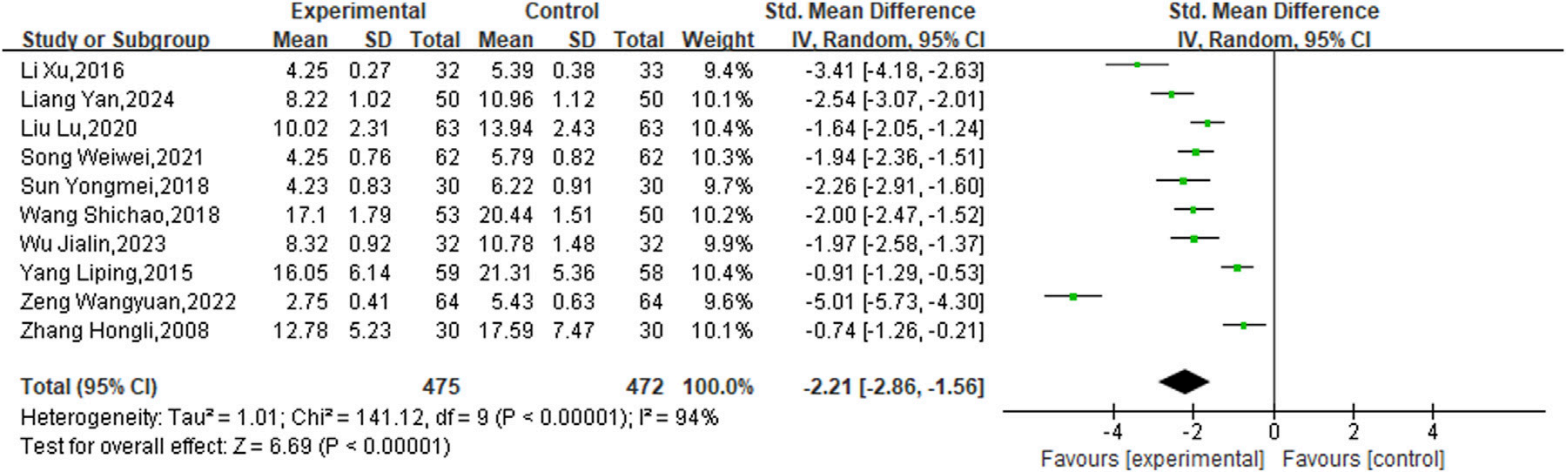
Meta result of NIHSS.

In addition, ten studies targeted the NIHSS specifically. The analysis was performed using a random-effects model after the heterogeneity test (P < 0.00001, I^2^ = 94%). The meta-analysis revealed a statistically significant disparity between the two groups, indicating that the experimental groups was more effective in enhancing NIHSS scores than the control groups (SMD = −2.21, 95%CI [−2.86, −1.56], P < 0.00001) (Figure 8).

#### 3.3.7 Blood lipid indicators

**FIGURE 9:**
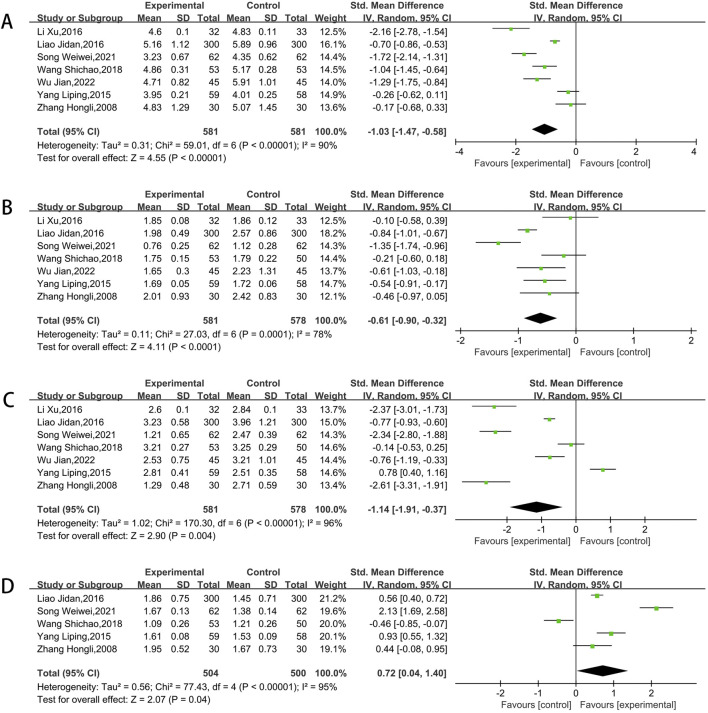
Meta result of TC **(A)**,TG **(B)**, LDL-C **(C)**, HDL-C **(D)**.

Among the included studies, seven articles reported on total cholesterol (TC), triglyceride (TG), and low-density lipoprotein cholesterol (LDL-C), while five articles reported on high-density lipoprotein cholesterol (HDL-C). The process involved heterogeneity testing, followed by conducting a meta-analysis with the random-effects model. The outcomes revealed that, the experimental groups was related to a reduction in TC (SMD = −1.03, 95% CI [-1.47, −0.58]; P < 0.00001) (Figure 9A), a reduction in TG (SMD = −0.61, 95% CI [-0.90, −0.32]; P < 0.00001) (Figure 9B), a reduction in LDL-C (SMD = −1.14, 95% CI [-1.91, −0.37]; P = 0.0002) (Figure 9C), as well as a greater increase in HDL-C(SMD = 0.72, 95%CI [0.04, 1.40]; P < 0.00001) (Figure 9D).

#### 3.3.8 Hemorheology indicators

**FIGURE 10:**
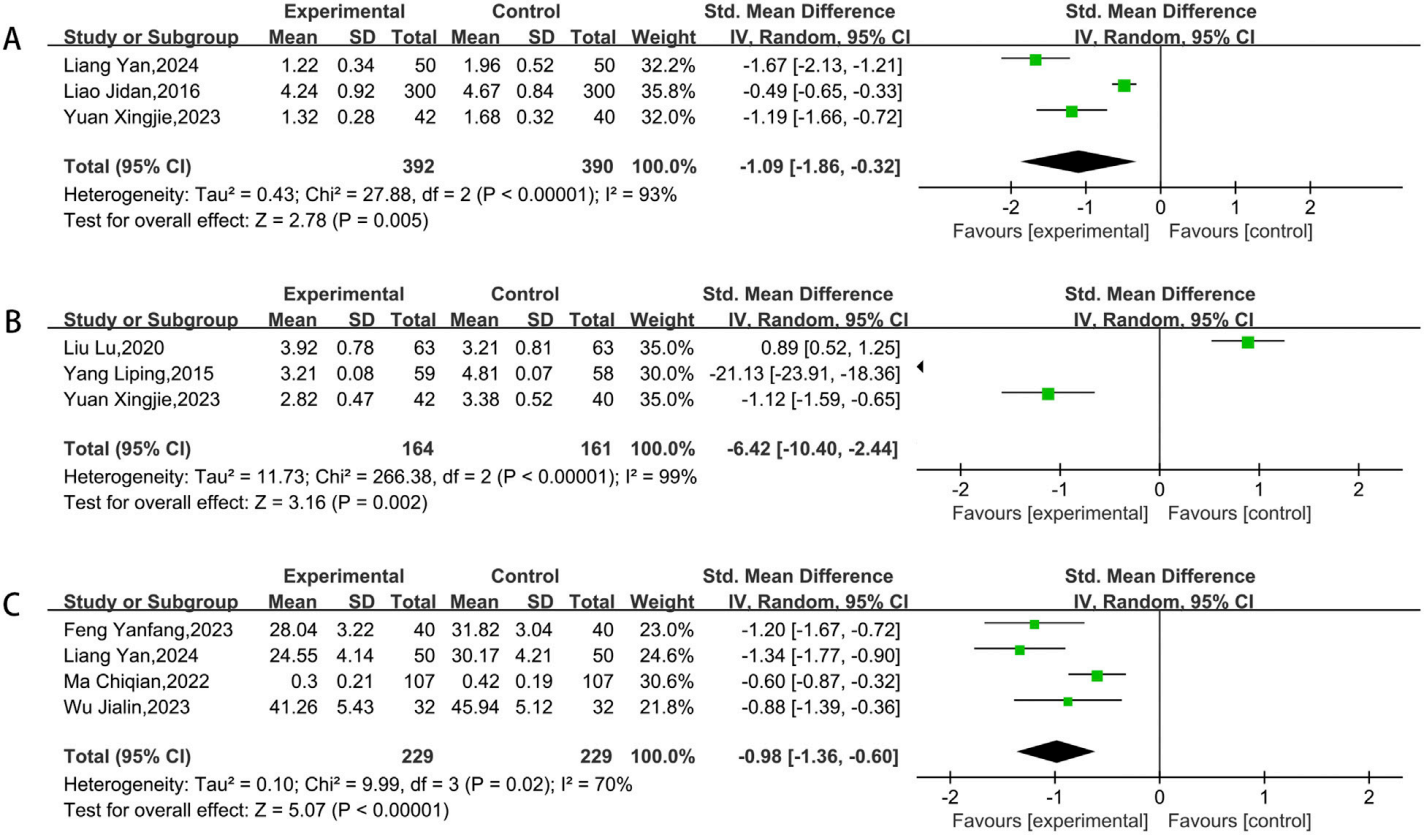
Meta result of blood viscosity **(A)**, fibrinogen **(B)**, platelet aggregation **(C)**.

A total of three studies assessed blood viscosity and fibrinogen, while four studies examined platelet aggregation. After conducting heterogeneity testing, a meta-analysis using the random-effects model was performed. The findings showed that the experimental groups was more effective in lowering blood viscosity (SMD = −1.09, 95% CI [-1.86,-0.32]; P < 0.00001) (Figure 10A), fibrinogen (SMD = −6.42, 95% CI [-10.40,-2.44]; P < 0.00001) (Figure 10B) and platelet aggregation (SMD = −0.98, 95% CI [-1.36,-0.60]; P < 0.00001) (Figure 10C) compared to control groups.

#### 3.3.9 Adverse reaction

**FIGURE 11:**
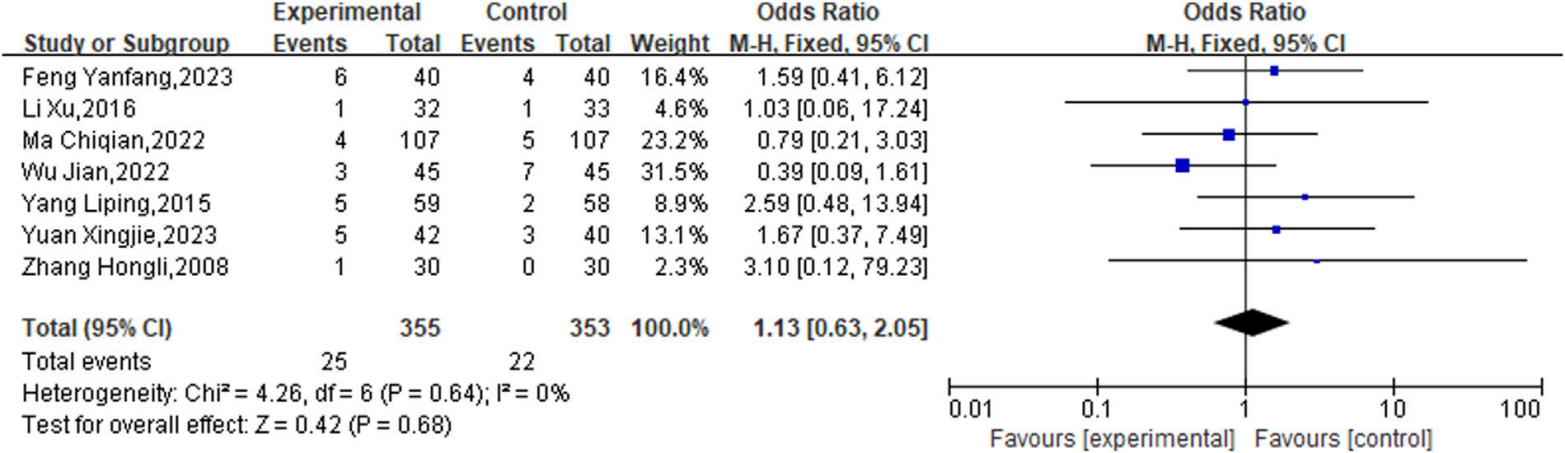
Meta result of Adverse reaction.

The occurrence of adverse reactions was referenced in seven studies from the literature. During treatment, 25 patients in the experimental groups had adverse reactions, including 12 instances of gastrointestinal issues, 4 instances of allergic reactions, 3 instances of bleeding, 3 instances of hematuria, 2 instances of skin redness, and 1 instance of dizziness. Among the control groups, 22 patients reported adverse reactions, with 5 instances experiencing gastrointestinal discomfort, 4 instances having hematuria, 3 instances feeling dizzy, 3 instances suffering from hemorrhage, 2 instances experiencing palpitations, 2 instances having headaches, and 1 instance of eryth. There was no overall heterogeneity detected, supporting the application of the fixed-effects model (P = 0.64, I2 = 0%). According to the meta-analysis data, the differences lacked statistical significance. (OR = 1.13, 95% CI [0.63, 2.05], P = 0.68) (Figure 11). The result indicates that the safety of administering medication was equivalent in the experimental and control groups, meaning that the use of CHM alongside conventional western medicine treatments did not result in more adverse reactions.

#### 3.3.10 Sensitivity analysis and publication bias

**FIGURE 12:**
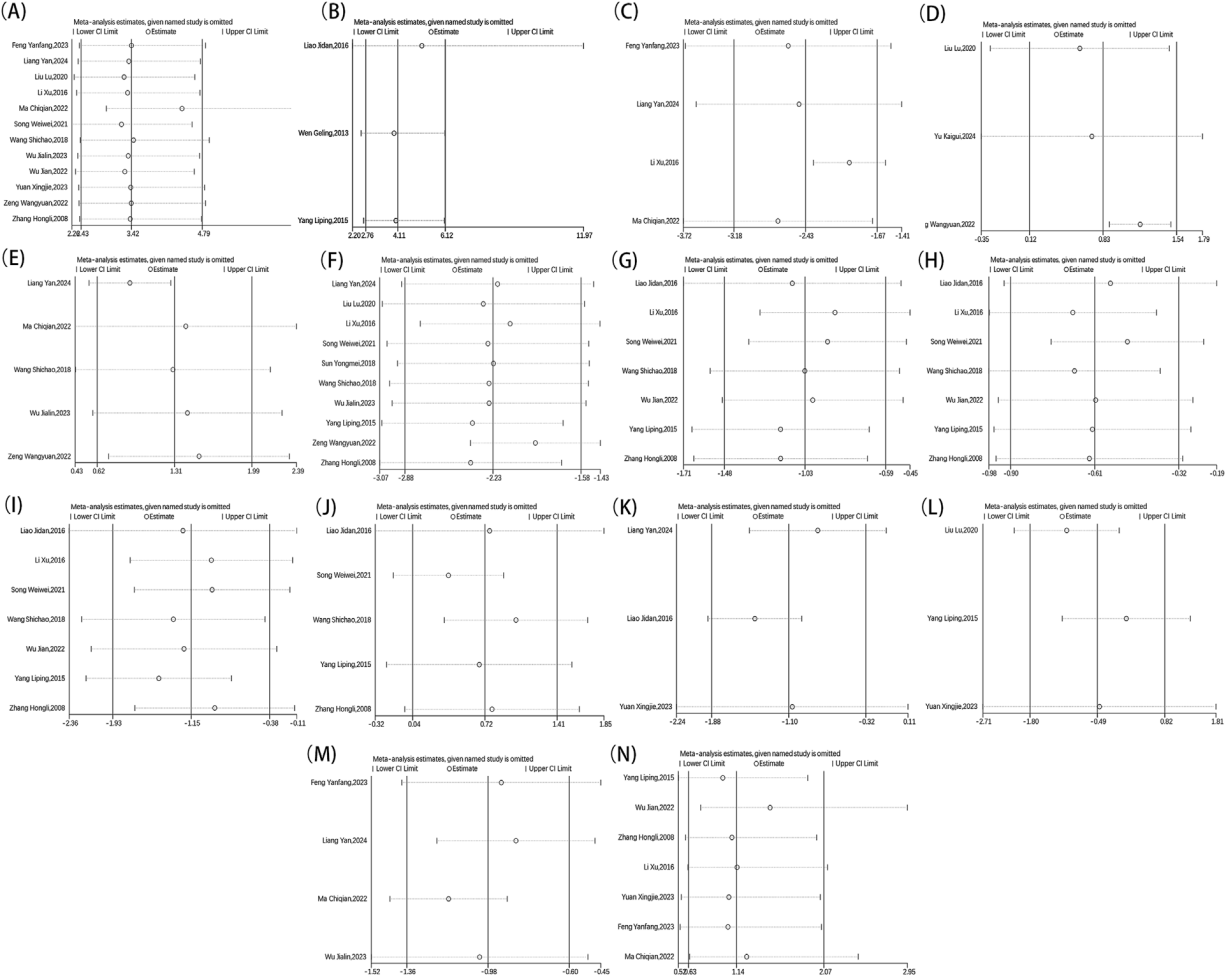
Metaninf result of overall effective rate **(A)**, ECG performance **(B)**, TCM scores **(C)**, LVEF **(D)**, MMSE **(E)**, NIHSS **(F)**, TC **(G)**, TG **(H)**, LDL-C **(I)**, HDL-C **(J)**, blood viscosity **(K)**, fibrinogen **(L)**, platelet aggregation **(M)**, adverse reaction **(N)**.

Each outcome obtained from the chosen literature underwent a detailed item-by-item elimination process to perform a sensitivity analysis. Finally, no significant changes were detected in the stability of individual studies or the aggregated effect sizes, thereby supporting the reliability of the data analysis results (Figure 12).

**FIGURE 13:**
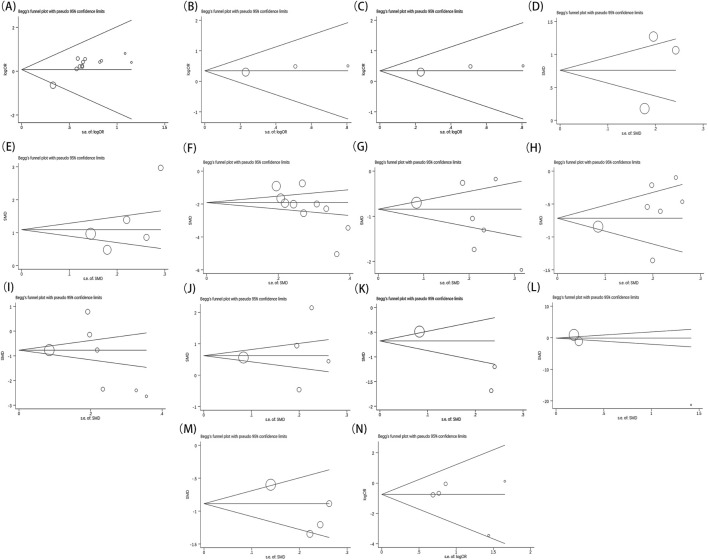
Begg’s test result of overall effective rate **(A)**, ECG performance **(B)**, TCM scores **(C)**, LVEF **(D)**, MMSE **(E)**, NIHSS **(F)**, TC **(G)**, TG **(H)**, LDL-C **(I)**, HDL‐C **(J)**, blood viscosity **(K)**, fibrinogen **(L)**, platelet aggregation **(M)**, adverse reaction **(N)**.

**FIGURE 14:**
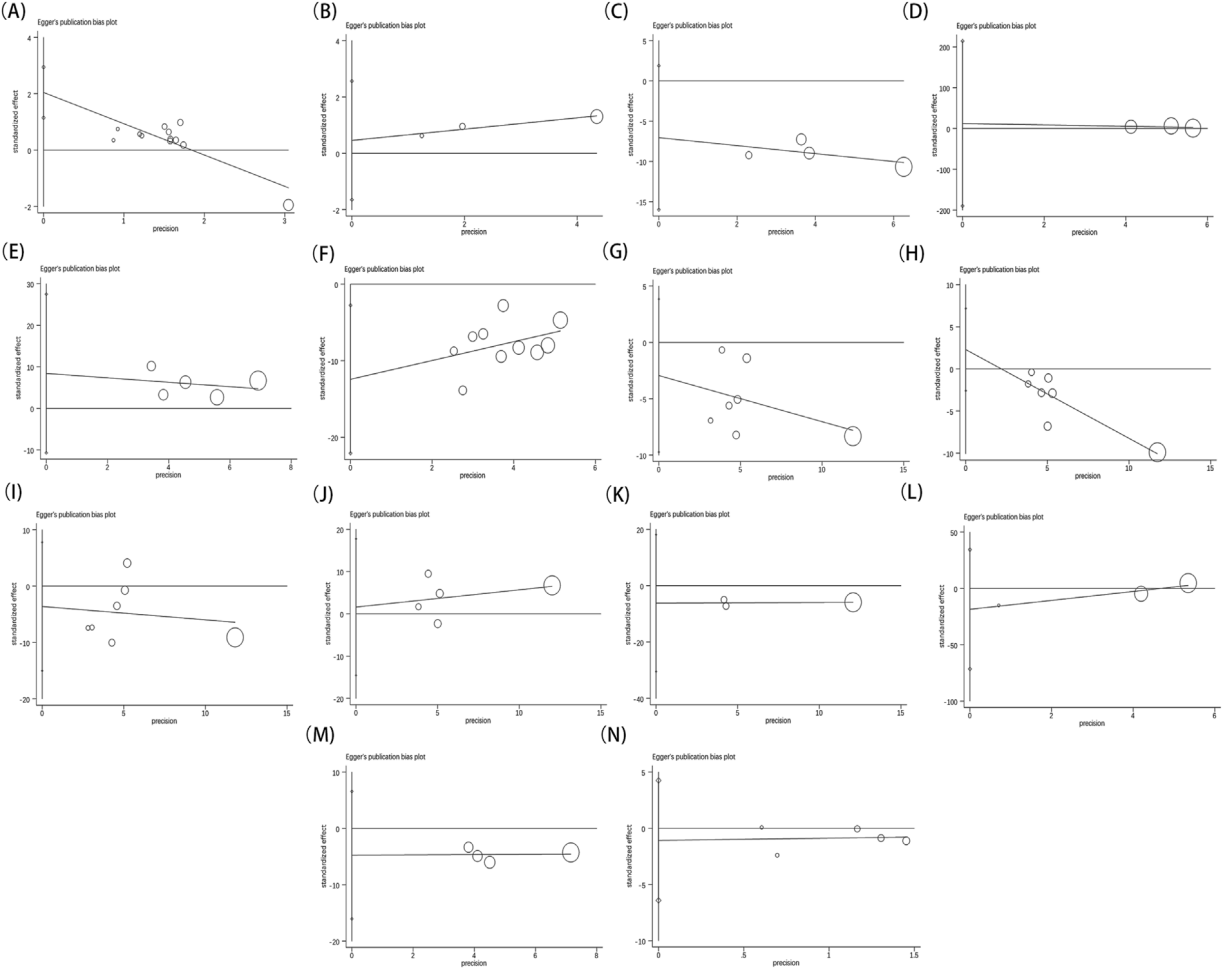
Egger’s test result of overall effective rate **(A)**, ECG performance **(B)**, TCM scores **(C)**, LVEF **(D)**, MMSE **(E)**, NIHSS **(F)**, TC **(G)**, TG **(H)**, LDL-C **(I)**, HDL‐C **(J)**, blood viscosity **(K)**, fibrinogen **(L)**, platelet aggregation **(M)**, adverse reaction **(N)**.

Furthermore, each outcome was subjected to Begg’s (Figure 13)and Egger’s (Figure 14) tests to check for publication bias, where a p-value below 0.05 signified its presence. No publication bias was found in the analysis for indicators besides the overall effective rate and NIHSS. These findings were thoroughly detailed in Table 2. Since all the studies were carried out in China and yielded positive results, we theorized that the publication bias observed might be influenced by the geographical area, racial demographics, and the omission of negative findings.

**TABLE 2:** Overview of sensitivity analysis and publication bias.

Indicators	OR/MD fluctuations	95%CI fluctuations	Publication bias (*P* value)	
			Begg’s test	Egger’s test
Overall effective rate	3.42	(2.43, 4.79)	0.115	0.000
ECG	4.11	(2.76, 6.12)	1.000	0.222
TCM syndrome score	−2.42	(−3.18, −1.67)	0.308	0.077
LVEF	0.83	(0.71, 1.27)	1.000	0.581
MMSE	1.31	(0.62, 1.99)	0.221	0.256
NIHSS	−2.23	(−2.88, −1.57)	0.007	0.018
TC	−1.03	(−1.48, −0.59)	0.368	0.316
TG	−0.61	(−0.90, −0.32)	0.548	0.218
LDL-C	−1.15	(−1.93, −0.38)	0.133	0.453
HDL-C	0.72	(0.37, 1.41)	0.806	0.772
Blood viscosity	−1.10	(−1.88, −0.32)	1.000	0.191
FIB	−0.49	(−1.80, 0.82)	0.296	0.141
Platelet aggregation	−0.97	(−1.35, −0.60)	1.000	0.213
Adverse reaction	1.14	(0.63, 2.07)	0.806	0.563

### 3.4 Quality of evidence

The evidence quality was rated using the GRADE system, with outcomes ranging from moderate to poor due to potential bias and imprecision in the findings. Detailed information regarding each outcome is provided in Table 3.

**TABLE 3:** GRADE evidence quality of outcomes included in the literature.

Quality assessment							No. of patients		Effect		Quality	Importance
No. of studies	Design	Risk of bias	Inconsistency	Indirectness	Imprecision	Other considerations	Experimental	Control	Relative	Absolute		
*Total Effective Rate*												
14	RCT	Serious^a^	No serious inconsistency	No serious indirectness	No serious imprecision	None	566/620 (91.3%)	467/616 (75.8%)	OR 3.42 (2.43–4.79)	157 more per 1,000 (from 126 more to 179 more)	Moderate	Critical
								75.5%		158 more per 1,000 (from 127 more to 182 more)		
*ECG*												
3	RCT	Serious^a^	No serious inconsistency	No serious indirectness	Serious^b^	None	366/405 (90.4%)	282/402 (70.1%)	OR 4.11 (2.76–6.12)	205 more per 1,000 (from 165 more to 233 more)	Low	Important
								70%		206 more per 1,000 (from 166 more to 235 more)		
*TCM scores*												
4	RCT	Serious^a^	No serious inconsistency	No serious indirectness	Serious^b^	None	229	230	-	SMD 2.4 lower (3.5–1.66 lower)	Low	Important
*LVEF*												
3	RCT	Serious^a^	No serious inconsistency	No serious indirectness	Serious^b^	None	166	166	-	SMD 0.82 higher (0.12–1.53 higher)	Low	Important
*MMSE*												
5	RCT	Serious^a^	No serious inconsistency	No serious indirectness	Serious^b^	None	306	303	-	SMD 1.3 higher (0.62–1.98 higher)	Low	Important
*NIHSS*												
10	RCT	Serious^a^	No serious inconsistency	No serious indirectness	No serious imprecision	None	475	472	-	SMD 2.21 lower (2.86–1.56 lower)	Moderate	Important
*TC*												
7	RCT	Serious^a^	No serious inconsistency	No serious indirectness	No serious imprecision	None	581	578	-	SMD 1.03 lower (1.47–0.58 lower)	Moderate	Important
TG												
7	RCT	Serious^a^	No serious inconsistency	No serious indirectness	No serious imprecision	None	581	578	-	SMD 0.61 lower (0.9–0.32 lower)	Moderate	Important
LDL-C												
7	RCT	Serious^a^	No serious inconsistency	No serious indirectness	No serious imprecision	None	581	578	-	SMD 1.14 lower (1.91–0.37 lower)	Moderate	Important
HDL-C												
7	RCT	Serious^a^	No serious inconsistency	No serious indirectness	No serious imprecision	None	581	578	-	SMD 0.72 higher (0.04–1.4 higher)	Moderate	Important
Blood viscosity												
3	RCT	Serious^a^	No serious inconsistency	No serious indirectness	Serious^b^	None	392	390	-	SMD 1.09 lower (1.86–0.32 lower)	Low	Important
Fibrinogen												
3	RCT	Serious^a^	No serious inconsistency	No serious indirectness	Serious^b^	None	164	161	-	SMD 6.42 lower (10.4–2.44 lower)	Low	Important
PA												
4	RCT	Serious^a^	No serious inconsistency	No serious indirectness	Serious^b^	None	229	229	-	SMD 0.98 lower (1.36–0.6 lower)	Low	Important
*Adverse Reaction*												
7	RCT	Serious^a^	No serious inconsistency	No serious indirectness	Serious^b^	None	25/355 (7%)	22/353 (6.2%)	OR 1.13 (0.63–2.05)	8 more per 1,000 (from 22 fewer to 58 more)	Low	Critical
								4.7%		6 more per 1,000 (from 17 fewer to 45 more)		

^a^ Details on certain randomization methods, allocation concealment, and blinding are lacking.

^b^ Included articles and observers were fewer in number.

### 3.5 Frequency analysis of CHM

A comprehensive total of 41 Chinese medicinal substances were utilized across all formulations. Table 4 enumerated those substances that appeared with a frequency of five or more instances. Notably, Salvia miltiorrhiza Bunge, Carthamus tinctorius L., and Ligusticum chuanxiong Hort. Were identified as the top three most frequently occurring substances.

**TABLE 4:** Frequency of CHMs (≥5 times).

Accepted name	Chinese name	Family	Number of studies
Salvia miltiorrhiza Bunge	Dan Shen	Lamiaceae	10
Carthamus tinctorius L	Hong Hua	Asteraceae	10
Ligusticum chuanxiong Hort	Chuan Xiong	Apiaceae	8
Angelica sinensis (Oliv.) Diels	Dang Gui	Apiaceae	6
Hirudo niponica Whitman	Shui Zhi	Hirudo	6
Citrus reticulata Blanco	Chen Pi	Rutaceae	5
Paeonia veitchii Lynch	Chi Shao	Paeoniaceae	5
Buthus martensii Karsch	Quan Xie	Scorpionida	5
Prunus persica (L.) Batsch	Tao Ren	Rosaceae	5

## 4 Discussion

### 4.1 Summary of evidence

A meta-analysis, incorporating 18 studies that met the established inclusion criteria, was conducted with a cumulative sample size of 2,202 participants. The study revealed that the utilization of CHM offered better outcomes than conventional western medicine treatment alone in treating CHD patients with CIS, demonstrated by higher overall effectiveness, improved ECG performance, and lower TCM syndrome scores. In clinical practice, LVEF is the most commonly utilized parameter for evaluating cardiac systolic function (Cohn et al., 2000). Beyond its primary role, LVEF also serves as an indicator of cerebral infarction severity and has been identified as a risk factor for cerebral infarction (Liu et al., 2021; McAreavey et al., 2010). Research indicates a negative correlation between LVEF levels in CIS patients and both the NIHSS and the modified Rankin Scale (mRS) (Tan et al., 2021). Consequently, we assessed LVEF as an outcome measure, and confirmed that the CHM offered advantages in improving cardiac function compared to solely using conventional western medicine treatment. The NIHSS score is an instrument employed to objectively measure stroke impairment and is the most frequently utilized scale for assessing stroke outcomes (Fischer et al., 2005). The severity of a stroke, as determined by the NIHSS score, is significantly correlated with mortality following an ischemic stroke (Ghabaee et al., 2014). The MMSE is routinely employed as a screening tool to evaluate cognitive function in patients experienced stroke, with higher scores on the MMSE indicating superior cognitive performance (Ismail et al., 2010). In our study, we utilized both NIHSS and MMSE as composite metric to facilitate the evaluation of neurological damage. Synthesizing the above results, our findings demonstrated that the CHM therapy provided notable advantages in reducing nervous system damage. In parallel, dyslipidemia is likewise a primary independent risk factor for CHD and CIS (Lee et al., 2017). Empirical evidence indicates that the reduction in HDL-C levels, coupled with the elevation in LDL-C levels, is significantly associated with the development of atherosclerotic plaque, thereby elevating the risk of cardiovascular and cerebrovascular diseases (Carmena et al., 2004; Kontush and Chapman, 2006). Concurrently, increased concentrations of cholesterol and triglycerides may facilitate lipid accumulation within the vascular wall, contributing to plaque formation and subsequently heightening the likelihood of arterial stenosis and cardiovascular events. Moreover, it is associated with heightened blood viscosity, platelet activation, and the encouragement of thrombogenesis, potentially obstructing cerebral blood flow (Kim et al., 2023). Thus, we assessed the alternation of HDL-C, LDL-C, TC and TG in the present meta-analysis, the result of which demonstrated that the CHM notably raised HDL-C levels while also improving LDL-C, TC, and TG in CHD patients with CIS, suggesting that this therapy is advantageous for enhancing blood lipid profiles. Blood viscosity and fibrinogen are indicative of endogenous coagulation function in patients, actively participating in the coagulation process and serving as significant predictors of cardiovascular events (Grotemeyer et al., 2014; Furukawa et al., 2016; Kowal and Marcinkowska-Gapińska, 2007; Aono et al., 2007). Simultaneously, CIS patients exhibit a range of hematological abnormalities, predominantly characterized by a hypercoagulable state, which is evidenced by increased blood viscosity, fibrinogen, and platelet aggregation (Chang and Jensen, 2014). Based on aforementioned evidences, we explored the alternation of hemorheology, and confirmed the potential of CHM therapy in adjusting blood viscosity, FIB, and platelet aggregation, with statistically significant. From a safety perspective, the CHM did not heighten the likelihood of adverse reactions.

### 4.2 Implications for practice

Salvia Miltiorrhiza Bunge, commonly known as Dan Shen in China, is extensively utilized in TCM for the treatment of cardiovascular diseases and ischemic stroke, owing to its multiple active constituents. Of these, danshensu is recognized as a principal vasodilatory agent in Salvia Miltiorrhiza Bunge. Research indicates that danshensu facilitates vasodilation by upregulating cyclooxygenase-2 (COX-2) expression and enhancing prostacyclin (PGI2) production (Wang et al., 2013). Moreover, danshensu confers cardioprotective effects by activating the Nrf2/HO-1 signaling pathway and interacting with the PI3K/Akt signaling pathway (Li X et al., 2016). Additionally, Tanshinone IIIA, another principal constituent of Salvia Miltiorrhiza Bunge, has been shown to reduce monocyte adhesion to vascular endothelial cells by inhibiting TNF-α-induced expression of adhesion molecules, thus exerting an anti-atherosclerotic effect (Chang et al., 2014). This mechanism may be pivotal in the management of CHD and CIS, as atherosclerosis is a leading etiological factor in these conditions. Concurrently, the water-soluble constituents of Salvia Miltiorrhiza Bunge, including danshensu and salvianolic acid B, have been demonstrated to activate the AKT signaling pathway, inhibit apoptotic processes, and thereby confer neuroprotective effects in cases of ischemic brain injury (Kim et al., 2020). This mechanism may mitigate neurological damage and functional deficits subsequent to ischemic stroke. Furthermore, the combined use of Salvia Miltiorrhiza Bunge and Ligusticum chuanxiong Hort. Has exhibited synergistic effects in the treatment of ischemic stroke. Empirical studies have indicated that this combination can substantially ameliorate brain infarction size, cerebral edema, and neurological function scores in stroke models, attributed to the interaction of their chemical constituents (Pang et al., 2024). In conclusion, Salvia Miltiorrhiza Bunge offers protective benefits in both coronary heart disease and ischemic stroke through diverse mechanisms, thus providing a scientific foundation for the therapeutic application of danshen in these conditions.

The principal active constituents of Carthamus tinctorius L., namely, hydroxyhipharanthin A (HSYA) and dehydrated hipharanthin B (ASYB), are recognized as the primary bioactive components in various hibiscus formulations (Qu et al., 2016). Research indicates that HSYA exerts multiple pharmacological effects pertinent to the treatment of coronary heart disease, including anti-inflammatory, antioxidant, and apoptosis-regulating activities (Zhou et al., 2018). Furthermore, the active constituents in safflower contribute directly to the activation of blood circulation and enhance their overall biological efficacy when used in conjunction with other herbs. For instance, the combination of safflower with ginseng and angelica has demonstrated significant anti-platelet and anticoagulant effects, thereby ameliorating CHD symptoms (Qu et al., 2016). The pharmacological benefits of safflower extend beyond the cardiovascular system, as it also exhibits neuroprotective properties. Studies have demonstrated that HSYA can ameliorate cognitive dysfunction induced by cerebral ischemia-reperfusion injury by restoring synaptic plasticity in the hippocampus (Yu et al., 2018).

Ligusticum chuanxiong Hort. Enhances blood circulation and decreases blood viscosity, thereby mitigating myocardial ischemia and exerting cardioprotective effects (Zeng et al., 2021). Research indicates that Ligusticum chuanxiong Hort. Also reduces the risk of cardiovascular events by modulating lipid profiles and inhibiting platelet aggregation (Parker et al., 2020). Furthermore, Ligusticum chuanxiong Hort. Has demonstrated efficacy in the secondary prevention of cardiovascular diseases. When combined with lifestyle modifications, it contributes to a reduction in the incidence and mortality associated with cardiovascular conditions (Shen et al., 2021). Key phytochemical constituents of Ligusticum chuanxiong Hort., such as ferulic acid and neocarboxylic acid, exhibit anti-inflammatory and antioxidant properties, as well as enhance blood circulation. These properties facilitate neuronal protection and support neuronal survival and growth (Zeng et al., 2021). Moreover, Ligusticum chuanxiong Hort. Augments its therapeutic impact on ischemic stroke by regulating blood pressure and preventing infections (Zeng et al., 2021).

Research has demonstrated that Angelica sinensis (Oliv.) Diels plays a role in modulating microglial cell polarization and the expression of neurosteroid receptors, potentially facilitating neurological function recovery post-stroke (Qin et al., 2023). The active constituents of Prunus persica (L.) Batschhas shown promise in inhibiting tissue factor expression and mitigating atherosclerosis, which is crucial for the prevention of cardiovascular diseases (Hao et al., 2019).

Meanwhile, according to our research, Hirudo niponica Whitman, Citrus reticulata Blanco, Paeonia veitchii Lynch., and Buthus martensii Karsch may play an important role in treating CHD patients with CIS. Above-mentioned findings suggest that Chinese herbal medicine possesses diverse potential and applicability in the treatment of cardiovascular and cerebrovascular diseases, offering both theoretical support and a practical foundation for their use in such medical contexts.

### 4.3 Strengths and limitations

Our study constitutes the first endeavor to quantitatively evaluate the beneficial effects of the CHM therapy on CHD complicated by CIS, providing a more rigorous evidence-based assessment for researchers globally. Our study’s main goal, in accordance with the Cochrane Collaboration guidelines, is to generate more detailed and definitive findings. The integration of additional outcome measures, including cardiac function indicators, blood pressure, and safety metrics, enables a thorough and multifaceted assessment of the efficacy of CHM therapy in the treatment of CHD complicated by CIS. The results of our study have significantly advanced understanding in this domain and have offered guidance for future research endeavors.

Nevertheless, it was crucial to recognize certain potential limitations despite our comprehensive evaluation of the existing evidence. Foremost among these was the general deficiency in methodological rigor observed in the included studies, primarily attributable to insufficient reporting of randomization procedures, allocation concealment, and blinding techniques. In particular, the implementation of blinding poses a significant challenge when both herbal decoction and conventional western medicine are employed as interventions, due to their markedly distinct external characteristics. Secondly, the presence of several confounding variables, including various interventions in the control group, might have led to the observed significant heterogeneity. Moreover, it was crucial to acknowledge that all the articles included in this review were published in Chinese and conducted within China. Fourth, the majority of studies incorporated into our analysis featured small sample sizes, which may have resulted in an overestimation of the intervention effects. Fifth, there was a notable lack of research regarding follow-up data on combination therapy in management.

### 4.4 Implications for research

In light of the aforementioned limitations, several recommendations are proposed for future research endeavors. Firstly, there is an urgent need for rigorously designed trials with high methodological quality. It is recommended that RCTs addressing CHD complicated by CIS be designed and reported in strict accordance with the CONSORT 2010 guidelines (Schulz et al., 2010) and the CONSORT Extension for Chinese Herbal Medicine Formulas 2017 (Cheng et al., 2017). Key methodological elements such as random sequence generation, allocation concealment, and blinding should be meticulously implemented in forthcoming studies. Secondly, it is imperative to validate our findings to evaluate the generalizability of TCM across broader and more diverse populations, encompassing various countries and ethnicities. Thirdly, considering the chronic and progressive nature of CHD complicated by CIS, which may fluctuate over time, continuous follow-up assessments are essential for accurately appraising both its immediate and long-term efficacy. Finally, it is important to recognize that this study predominantly relies on literature reviews and database analyses. Therefore, further experimental and clinical trials are necessary to substantiate the specific conclusions drawn.

## 5 Conclusion

Chinese herbal medicine demonstrated superior efficacy in the treatment of CHD patients with CIS. Concurrently, the Chinese herbal medicine therapy showed potential for improving neurological damage, lipid profiles, and positively affecting hemorheological parameters, all while minimizing the risk of adverse effects, thereby offering a robust foundation for the development of clinical guidelines. A thorough examination of combination therapy within the realm of cardio-cerebrovascular disease substantially aids in the preservation of TCM clinical knowledge, augments the comprehension of TCM theory and practice in the treatment of cardio-cerebrovascular conditions, and broadens the spectrum of therapeutic options available.

## Data Availability

The original contributions presented in the study are included in the article/Supplementary Material, further inquiries can be directed to the corresponding authors.
