# Supplementary material for: Therapeutic potential of Chinese herbal medicine for coronary heart disease patients with cerebral ischemic stroke: a systematic review and meta-analysis
Source: Front Pharmacol. 2025 Jul 16;16:1578783. doi: 10.3389/fphar.2025.1578783 (PMC12307363; doi:10.3389/fphar.2025.1578783)
Supplement: Supplementary file 1 [file DataSheet1.docx]

**Details of Search Strategy**

**Source: PubMed**

| Search | Query |
| --- | --- |
| #1 | “Coronary Heart Disease Complicated by Cerebral Ischemic Stroke” [Mesh] |
| #2 | Coronary Heart Disease Complicated by Cerebral Ischemic Stroke [Title/Abstract] |
| #3 | Coronary Heart Disease Complicated by Stroke [Title/Abstract] |
| #4 | CHD Complicated by CIS [Title/Abstract] |
| #5 | CHD Complicated by IS [Title/Abstract] |
| #6 | #1 OR #2 OR #3 OR #4 OR #5 |
| #7 | Chinese herbal medicine [Title/Abstract] |
| #8 | Chinese traditional [Title/Abstract] |
| #9 | Oriental traditional [Title/Abstract] |
| #10 | Traditional Chinese medicine [Title/Abstract] |
| #11 | Traditional Chinese medicinal materials [Title/Abstract] |
| #12 | Chinese herb* [Title/Abstract] |
| #13 | Herbal medicine [Title/Abstract] |
| #14 | Herbal decoction [Title/Abstract] |
| #15 | Tang [Title/Abstract] |
| #16 | Pill [Title/Abstract] |
| #17 | Wan [Title/Abstract] |
| #18 | Powder [Title/Abstract] |
| #19 | Formula [Title/Abstract] |
| #20 | Granule [Title/Abstract] |
| #21 | Capsule [Title/Abstract] |
| #22 | Particles [Title/Abstract] |
| #23 | Ointment [Title/Abstract] |
| #24 | Prescription [Title/Abstract] |
| #25 | Receipt [Title/Abstract] |
| #26 | #7 OR #8 OR #9 OR #10 OR #11 OR #12 OR #13 OR #14 OR #15 OR #16 OR #17 OR #18 OR #19 OR #20 OR #21 OR #22 OR #23 OR#24 OR #25 OR #26 OR #27 OR #28 OR#29 OR#30 OR#31 |
| #27 | Random controlled trial [Title/Abstract] |
| #28 | RCT [Title/Abstract] |
| #29 | Random [Title/Abstract] |
| #30 | Placebo [Title/Abstract] |
| #31 | #33 OR #34 OR #35 OR #36 |
| #32 | #6 AND #26 AND #31 |

**Source: Cochrane Library**

| Search | Query |
| --- | --- |
| #1 | MeSH descriptor: [Coronary Heart Disease Complicated by Cerebral Ischemic Stroke] explode all trees |
| #2 | (Coronary Heart Disease Complicated by Cerebral Ischemic Stroke): ti,ab,kw |
| #3 | (Coronary Heart Disease Complicated by Stroke): ti,ab,kw |
| #4 | (CHD Complicated by CIS): ti,ab,kw |
| #5 | (CHD Complicated by IS): ti,ab,kw |
| #6 | #1 OR #2 OR #3 OR #4 OR #5 |
| #7 | (Chinese herbal medicine): ti,ab,kw |
| #8 | (Chinese traditional): ti,ab,kw |
| #9 | (Oriental traditional): ti,ab,kw |
| #10 | (Traditional Chinese medicine): ti,ab,kw |
| #11 | (Traditional Chinese medicinal materials): ti,ab,kw |
| #12 | (Chinese herb*): ti,ab,kw |
| #13 | (Herbal medicine): ti,ab,kw |
| #14 | (Herbal decoction): ti,ab,kw |
| #15 | (Tang): ti,ab,kw |
| #16 | (Pill): ti,ab,kw |
| #17 | (Wan): ti,ab,kw |
| #18 | (Powder): ti,ab,kw |
| #19 | (Formula): ti,ab,kw |
| #20 | (Granule): ti,ab,kw |
| #21 | (Capsule): ti,ab,kw |
| #22 | (Particles): ti,ab,kw |
| #23 | (Ointment): ti,ab,kw |
| #24 | (Prescription): ti,ab,kw |
| #25 | (Receipt): ti,ab,kw |
| #26 | #7 OR #8 OR #9 OR #10 OR #11 OR #12 OR #13 OR #14 OR #15 OR #16 OR #17 OR #18 OR #19 OR #20 OR #21 OR #22 OR #23 OR#24 OR #25 OR #26 OR #27 OR #28 OR#29 OR#30 OR#31 |
| #27 | (Random controlled trial): ti,ab,kw |
| #28 | (RCT): ti,ab,kw |
| #29 | (Random): ti,ab,kw |
| #30 | (Placebo): ti,ab,kw |
| #31 | #33 OR #34 OR #35 OR #36 |
| #32 | #6 AND #26 AND #31 |

**Source: Embase**

| Search | Query |
| --- | --- |
| #1 | 'Coronary Heart Disease Complicated by Cerebral Ischemic Stroke '/exp |
| #2 | 'Coronary Heart Disease Complicated by Cerebral Ischemic Stroke': ab, ti |
| #3 | 'Coronary Heart Disease Complicated by Stroke': ab, ti |
| #4 | 'CHD Complicated by CIS': ab, ti |
| #5 | 'CHD Complicated by IS': ab, ti |
| #6 | #1 OR #2 OR #3 OR #4 OR #5 |
| #7 | 'Chinese herbal medicine': ab, ti |
| #8 | 'Chinese traditional': ab, ti |
| #9 | 'Oriental traditional': ab, ti |
| #10 | 'Traditional Chinese medicine': ab, ti |
| #11 | 'Traditional Chinese medicinal materials': ab, ti |
| #12 | 'Chinese herb': ab, ti |
| #13 | 'Herbal medicine': ab, ti |
| #14 | 'Herbal decoction': ab, ti |
| #15 | 'Tang': ab, ti |
| #16 | 'Pill': ab, ti |
| #17 | 'Wan': ab, ti |
| #18 | 'Powder': ab, ti |
| #19 | 'Formula': ab, ti |
| #20 | 'Granule': ab, ti |
| #21 | 'Capsule': ab, ti |
| #22 | 'Particles': ab, ti |
| #23 | 'Ointment': ab, ti |
| #24 | 'Prescription': ab, ti |
| #25 | 'Receipt': ab, ti |
| #26 | #7 OR #8 OR #9 OR #10 OR #11 OR #12 OR #13 OR #14 OR #15 OR #16 OR #17 OR #18 OR #19 OR #20 OR #21 OR #22 OR #23 OR#24 OR #25 OR #26 OR #27 OR #28 OR#29 OR#30 OR#31 |
| #27 | 'Random controlled trial): ab, ti |
| #28 | 'RCT): ab, ti |
| #29 | 'Random): ab, ti |
| #30 | 'Placebo): ab, ti |
| #31 | #33 OR #34 OR #35 OR #36 |
| #32 | #6 AND #26 AND #31 |

**Source: Web of Science**

| Search | Query |
| --- | --- |
| #1 | TS="Coronary Heart Disease Complicated by Cerebral Ischemic Stroke" |
| #2 | TS="Coronary Heart Disease Complicated by Cerebral Ischemic Stroke" |
| #3 | TS="Coronary Heart Disease Complicated by Stroke" |
| #4 | TS="CHD Complicated by CIS" |
| #5 | TS="CHD Complicated by IS" |
| #6 | #1 OR #2 OR #3 OR #4 OR #5 |
| #7 | TS="Chinese herbal medicine" |
| #8 | TS="Chinese traditional" |
| #9 | TS="Oriental traditional" |
| #10 | TS="Traditional Chinese medicine" |
| #11 | TS="Traditional Chinese medicinal materials" |
| #12 | TS="Chinese herb" |
| #13 | TS="Herbal medicine" |
| #14 | TS="Herbal decoction" |
| #15 | TS="Tang" |
| #16 | TS="Pill" |
| #17 | TS="Wan" |
| #18 | TS="Powder" |
| #19 | TS="Formula" |
| #20 | TS="Granule" |
| #21 | TS="Capsule" |
| #22 | TS="Particles" |
| #23 | TS="Ointment" |
| #24 | TS="Prescription" |
| #25 | TS="Receipt" |
| #26 | #7 OR #8 OR #9 OR #10 OR #11 OR #12 OR #13 OR #14 OR #15 OR #16 OR #17 OR #18 OR #19 OR #20 OR #21 OR #22 OR #23 OR#24 OR #25 OR #26 OR #27 OR #28 OR#29 OR#30 OR#31 |
| #27 | TS="Random controlled trial" |
| #28 | TS="RCT" |
| #29 | TS="Random" |
| #30 | TS="Placebo" |
| #31 | #33 OR #34 OR #35 OR #36 |
| #32 | #6 AND #26 AND #31 |

**CNKI**

(SU = '冠心病合并脑卒中' OR SU='冠心病合并中风' ) AND ( FT='中医' OR FT='中药' OR FT='中医药' OR FT='中西医' OR FT='中成药' OR FT='汤' OR FT='片' OR FT='丸' OR FT='散' OR FT='胶囊' OR FT='颗粒' OR FT='水' OR FT='液' OR FT='合剂' OR FT='注射液') AND (FT='随机')

**VIP**

(M=冠心病合并脑卒中 OR 冠心病合并中风) AND ((U=中医 OR 中药 OR 中西医OR中成药 OR 汤 OR 片 OR 丸 OR 散 OR 胶囊 OR 颗粒 OR水 OR液 OR 合剂 OR 注射液 ) OR (R=中医 OR 中药 OR 中西医OR中成药 OR 汤 OR 片 OR 丸 OR 散 OR 胶囊 OR 颗粒 OR水 OR液 OR 合剂 OR 注射液)) AND ((U=随机 ) OR (R=随机 ))

**Wanfang**

全部:(( "冠心病合并脑卒中" OR "冠心病合并中风") AND ( "中医" OR "中药" OR "中西医" OR "中成药" OR "汤" OR "片" OR "丸" OR "散" OR "胶囊" OR "颗粒" OR "水" OR "液" OR "合剂" OR "注射液" ) AND ("随机" )

**CBM**

(("冠心病合并脑卒中"[全部字段:智能] OR "冠心病合并中风"[全部字段:智能] )) AND(( "中医"[全部字段:智能] OR "中药"[全部字段:智能] OR "中西医"[全部字段: 智能] OR ("中成药"[常用字段:智能] OR "汤"[全部字段:智能]) OR "片"[全部字段:智能] OR "丸"[全部字段:智能] OR "散"[全部字段:智能] OR "胶囊"[全部字段:智能] OR "颗粒"[全部字段:智能] OR "水"[全部字段:智能] OR"液"[全部字段:智能] OR "合剂"[全部字段:智能] OR "注射液"[全部字段:智能])) AND ("随机"[全部字段:智能])
